# Normal recognition of famous voices in developmental prosopagnosia

**DOI:** 10.1038/s41598-020-76819-3

**Published:** 2020-11-12

**Authors:** Maria Tsantani, Richard Cook

**Affiliations:** Department of Psychological Sciences, Birkbeck, University of London, Malet Street, London, WC1E 7HX UK

**Keywords:** Psychology, Human behaviour

## Abstract

Developmental prosopagnosia (DP) is a condition characterised by lifelong face recognition difficulties. Recent neuroimaging findings suggest that DP may be associated with aberrant structure and function in multimodal regions of cortex implicated in the processing of both facial and vocal identity. These findings suggest that both facial and vocal recognition may be impaired in DP. To test this possibility, we compared the performance of 22 DPs and a group of typical controls, on closely matched tasks that assessed famous face and famous voice recognition ability. As expected, the DPs showed severe impairment on the face recognition task, relative to typical controls. In contrast, however, the DPs and controls identified a similar number of voices. Despite evidence of interactions between facial and vocal processing, these findings suggest some degree of dissociation between the two processing pathways, whereby one can be impaired while the other develops typically. A possible explanation for this dissociation in DP could be that the deficit originates in the early perceptual encoding of face structure, rather than at later, post-perceptual stages of face identity processing, which may be more likely to involve interactions with other modalities.

## Introduction

Developmental prosopagnosia (DP) is a neurodevelopmental condition characterized by lifelong face recognition difficulties that are present despite normal intelligence and low-level vision, and without brain damage^1–3^. Individuals with DP exhibit difficulties identifying familiar faces^4,5^ as well as impaired discrimination and matching of unfamiliar faces^6,7^. These perceptual difficulties impair the identification of individuals irrespective of face ethnicity^8^. Many individuals with DP show subtle impairments in the recognition of facial expressions^9^ and facial sex^10,11^. Some DPs also show impaired matching of human bodies^12^ and non-face objects^13,14^. Historically the condition was thought to be rare, however current estimates suggest that ~ 2% of the general population experience lifelong face recognition problems severe enough to disrupt their daily lives^15–17^.

Despite growing interest in DP, the cause of the condition remains unclear. It is well-established that DP runs in families, a finding that suggests the condition may have a genetic origin^18–22^. This view accords with evidence that face recognition is a heritable trait^23,24^. In the past, cognitive accounts have argued that DPs struggle to identify faces because individuals fail to derive an integrated “holistic” representation of different facial regions; instead, DPs may process faces using a piecemeal analysis^25,26^. However, recent findings that DPs exhibit behavioural markers of holistic face processing challenge this view^5,27^.

### Present study

The present study examined whether individuals with DP, who experience life-long face recognition difficulties, also exhibit impaired voice recognition. Several cortical regions implicated in the visual processing of facial identity also appear to be involved in the processing of vocal identity. In particular, regions of the anterior temporal lobe (ATL) have been implicated in the processing of identity from both faces^28–31^ and voices^32–35^. The ATL has been described as a “multimodal hub” for the recognition of person identity^36^, and is thought to mediate post-perceptual processing of face and voice identity^37,38^. Similarly, the posterior portion of the superior temporal sulcus (pSTS) responds to both faces and voices^39,40^, and forms modality-general person-identity representations that integrate information from the face and voice^41–43^.

Some individuals with acquired prosopagnosia (AP) exhibit impaired voice recognition in addition to their face recognition deficits^44–47^. In cases of AP, individuals develop typical face recognition ability during childhood and adolescence, but subsequently experience face recognition problems following a brain injury^48,49^. Where observed, co-occurring face and voice recognition deficits are often associated with damage to multimodal regions, notably the anterior temporal lobe (ATL)^37,47^. These findings lend support to the view that these multimodal regions make a causal contribution to both face and voice recognition.

Several studies suggest structural or functional atypicality within putative multimodal regions in DP^50–52^. Where observed, these differences are subtle, and there is currently little consensus on what parts of the brain are affected and how. Nevertheless, there is evidence of reduced activation to faces and reduced grey matter volume in regions of the ATL^50,51^, and reduced selectivity for faces in the pSTS^52^. The implication of multimodal brain regions in DP further suggests the possibility that voice recognition may also be affected.

Interactions between face and voice identity processing have also been demonstrated behaviourally. It has been shown that learning a voice alongside a face improves subsequent voice recognition in typical participants^53^. There is also evidence from cross-modal priming studies showing that the processing of familiar voices is facilitated after viewing the corresponding face, and vice versa^54–56^. These findings indicate that the processing of facial identity informs the processing of vocal identity, and vice versa. Thus, it is possible that impairment in one modality (e.g., the visual processing of faces) could affect identity recognition in the other modality (e.g., recognition of vocal identity).

Existing research has largely focused on the ability of DPs to discriminate and memorise unfamiliar voices. In one study of 12 DPs, all but one showed typical short-term memory for unfamiliar voices^57^. Employing similar tasks, a subsequent study of 12 DPs found that 3 individuals showed signs of a voice processing deficit^58^. These findings suggest that the majority of DPs show intact matching of unfamiliar voices, but that deficits may be present in some cases. Less is known about the ability of DPs to recognize familiar voices. To date, recognition of familiar voices has been examined in only one adult DP, who showed impaired recognition of personally familiar voices, despite showing typical performance in an unfamiliar voice recognition task^59^. Impaired recognition of personally familiar voices has also been described in a 5-year old child with severe DP^60^.

In the present study we sought to determine whether adults with DP show impaired recognition of celebrity voices. Famous face recognition tasks are thought to reveal the face processing problems in DP more effectively than unfamiliar face matching tasks^4^. Typical individuals are thought to have stored representations for thousands of familiar faces^61^. Recognising a particular famous face therefore poses the cognitive system with a formidable needle-in-a-haystack problem: only one of these stored representations matches the test stimulus. Solving this problem requires a precise representation of the to-be-identified face—a level of representational precision that DPs may struggle to achieve^5,6^. In contrast, an impoverished perceptual description may often be adequate to infer the correct solution when completing matching tasks with unfamiliar faces, where only one or two options need to be considered/rejected.

Applying the same logic to voice recognition, it is possible that tests of famous voice recognition may reveal voice recognition deficits in DP, that go undetected by unfamiliar voice matching tasks. It is also possible that some DPs have a selective deficit that impairs the recognition of familiar voices, but not the matching and discrimination of unfamiliar voices. The ATL is thought to contribute to the recognition of familiar faces and voices by encoding semantic knowledge, such as name and occupation^62–64^. Importantly, we accumulate semantic knowledge as individuals become more familiar. Little if any semantic knowledge is available for unfamiliar individuals. If DP affects brain systems that encode semantic knowledge, familiar voice identification could be impaired alongside famous face identification, while the perceptual processing of unfamiliar voices remains unaffected.

## Methods

### Online testing and participant recruitment

The experiment described was conducted online using Gorilla^65^. Participants completed the study on their personal computer or laptop. The use of online testing is increasingly common. Carefully-designed online tests of cognitive and perceptual processing can yield high-quality data, indistinguishable from that collected in the lab^66–68^.

Twenty-two individuals with DP (8 males, *M*_age_ = 39.73 years, *SD*_age_ = 13.65 years) and 44 typical controls (18 males, *M*_age_ = 36.57 years, *SD*_age_ = 8.23 years) took part in the study. The groups did not differ significantly in terms of age [*t*(28.854) = 0.998, *p* = 0.326, *d* = 0.280, CI_95%_ = − 0.258, 0.775] or the proportion of male participants [*X*^2^_(1)_ = 0.127, *p* = 0.723]. Sample size was determined *a-priori* based on similar group studies of DP^8,9,11,12,27,69^.

DP participants were recruited through https://www.troublewithfaces.org and reported face recognition difficulties in the absence of brain damage or neurological illness. Diagnostic decisions were based on participants’ scores on two versions of the Cambridge Face Memory Test (CFMT), the CFMT-original^7^ and the CFMT-Australian^70^, and on the Twenty-Item Prosopagnosia Index (PI20)^71,72^. DPs also completed the Cambridge Car Memory Test (CCMT)^73^ to assess their within-class object recognition ability. All diagnostic tests were completed online. Diagnostic information for each DP is provided in Table 1.

**Table 1:** Diagnostic information for the DP participants. *≤ 1SD from typical mean; **≤ 2SDs from typical mean; ***≤ 3SDs from typical mean.

	Age	Gender	PI20	CFMT	CFMT-A	CCMT
1	25	F	75***	50.0***	58.3**	76.4
2	26	M	76***	38.9***	58.3**	51.4*
3	55	F	82***	52.8***	56.9**	65.3
4	25	F	78***	54.2***	50.0***	72.2
5	49	M	76***	51.4***	63.9*	79.2
6	56	M	90***	54.3***	52.8**	86.1
7	64	M	68***	61.1**	59.7**	59.7*
8	24	F	89***	43.1***	37.5***	75.0
9	39	F	80***	51.4***	40.3***	66.7
10	30	F	69***	58.3***	61.1*	56.9*
11	27	F	79***	59.7**	63.9*	47.2**
12	49	M	85***	51.4***	44.4***	77.8
13	30	M	70***	54.2***	58.3**	59.7*
14	59	F	75***	61.1**	56.9**	59.7*
15	58	F	78***	34.7***	54.2**	55.6*
16	33	F	71***	51.4***	63.9*	79.2
17	46	F	79***	62.5**	50.0***	61.1*
18	21	F	76***	58.3***	54.2**	75.0
19	53	M	86***	40.3***	58.3**	75.0
20	35	F	72***	56.9***	56.9**	70.8
21	40	M	69***	48.6***	58.3**	86.1
22	30	F	85***	65.3**	54.2**	75.0
DP mean			77.6	52.7	55.1	68.7
DP SD			6.5	8.0	7.1	11.0
Comparison mean			38.0	85.0	80.2	73.5
Comparison SD			9.1	8.9	10.2	12.6

Nb. Comparison data (N = 54) for the PI20, and CFMT were taken from Biotti et al.^6^. Comparison data (N = 75) for the CFMT-A were taken from McKone et al.^11^. Comparison data (N = 61) for the CCMT were taken from Gray et al.^14^.

Control participants were recruited through Prolific (https://www.prolific.co), and were required to have an approval rating of 95%. Three control participants were replaced having scored more than 65 on the PI20. A score of 65 has been recommended as a cut-off for DP^71,72^. As expected, the PI20 scores of the control group (*M* = 43.16, *SD* = 9.16) were significantly lower compared with the DP group (*M* = 77.64, *SD* = 6.50) [*t*(64) = 15.752, *p* < 0.001, *d* = 4.065, CI_95%_ = 3.231, 4.983].

All participants were required to be between 20 and 65 years-old, to have normal or corrected-to-normal visual acuity and hearing, and to have had no clinical diagnosis of autism spectrum disorder. To ensure that participants would be familiar with the famous people whose faces and voices were presented in the tasks, participants were required to have English as their first language, and to have been resident in the UK for a minimum of 10 years (all except three participants, one in the control group and two in the DP group, had been resident in the UK their entire life). These inclusion criteria were identified at the outset.

Ethical clearance was granted by the Departmental Ethics Committee for Psychological Sciences, Birkbeck, University of London. The experiment was conducted in line with the ethical guidelines laid down in the 6th (2008) Declaration of Helsinki. All participants provided informed consent and were paid a small honorarium. The experimental tasks are available as Open Materials at gorilla.sc (https://gorilla.sc/openmaterials/115074). Data for the experimental tasks are available via the Open Science Framework (https://osf.io/da2xu/).

### Face and voice recognition tasks

Thirty images of celebrity faces were presented in a face recognition task, and 30 audio clips of celebrity voices were presented in a voice recognition task. Different celebrities were presented in the face and voice tasks. A complete list is provided in the supplementary materials (Table S1). These celebrities were chosen based on pilot studies showing that their face or voice were frequently recognized by British participants aged between 20 and 65. Celebrities were British or American and included singers, actors, models, royalty, politicians, athletes, and TV personalities. In each task, half of the celebrities were men and half were women. Within each task, stimulus order was randomised. The order of the face and voice tasks was counterbalanced across participants.

The 30 images used in the famous face recognition task were sourced though internet searches. Faces were front-facing and exhibited direct eye gaze and a neutral or smiling facial expression. Faces were cropped to an oval to exclude external features. The images were converted to grey-scale and equated for luminance using the SHINE toolbox^74^ in Matlab (The MathWorks, Natick, MA). Each trial began with a fixation cross presented for 250 ms, followed by a face presented for 5 s.

The 30 audio clips used in the famous voice recognition task were extracted from videos on https://www.youtube.com. The audio clips contained between 7–10 s of speech. The clips were converted to mono with a sampling rate of 44,100, low-pass filtered at 10 kHz, and root-mean-square (RMS) normalised in intensity using Praat^75^. The audio clips were selected so that the speakers could not be identified based on the speech content. Participants were asked to complete the task in a quiet environment where they could clearly hear sounds from their device, and were encouraged to wear headphones. Before starting the main task, participants were presented with an example audio clip which they could replay to adjust the volume on their device to a comfortable level. In each trial, participants were asked to click on a button to hear the audio clip. Each clip could be played up to three times.

In both tasks, a response screen asked participants to identify the person by typing their full name or other uniquely identifying information (e.g., a famous TV role or sporting achievement). Participants were also asked if the face or voice was familiar (Yes/No). To check that participants were paying attention, we also included a question about the gender of each face or voice (Woman/Man). When completing the face task, all participants completed the attention check correctly on at least 28 of the 30 trials (20 out of 22 DPs, and 38 out of 44 controls responded correctly on all trials). When completing the voice task, all participants completed the attention check correctly on at least 29 of the 30 trials (19 out of 22 DPs, and 41 out of 44 controls responded correctly on all trials).

### Name recognition and exposure frequency

After completing the famous face and voice tasks, participants were asked to indicate which celebrities they knew by name. Participants were presented with the names of the sixty celebrities whose face or voice was used in the study. Participants viewed the names one at a time, and were asked to indicate whether they knew the person (Yes/No). They were also asked to indicate how frequently they were exposed to that person’s face or voice using a six-point scale ranging from ‘never’ to ‘very frequently’. Participants were asked to respond ‘never’ if they had indicated that they didn’t know the person by name.

### Voice recognition questionnaire

To assess participants’ self-reported voice recognition ability, we constructed a voice recognition questionnaire. The scale included 16 statements regarding voice recognition ability (Table 2). For example: *‘It is difficult for me to tell two people apart by their voices alone’*. Participants indicated the degree to which they agreed or disagreed with each statement using a five-point scale ranging from ‘strongly disagree’ to ‘strongly agree’. The items were scored so that higher overall scores indicate poor perceived voice recognition ability, and lower scores indicate good perceived ability. Scores could range from 16 to 80.

**Table 2:** The statements comprising the voice questionnaire.

1. I would quickly recognize friends and family if I heard them speaking on the radio
2. My friends and family think that I am bad at recognizing other people’s voices
3. It is easy to recognize celebrity voiceovers on television adverts
4. Failure to recognize a familiar voice has caused me embarrassment
5. If a friend called me from an unknown telephone number, I would quickly identify them from their voice alone
6. It is difficult for me to tell two people apart by their voices alone
7. I have excellent memory for the voices of other people
8. Other people often recognize voices that I cannot
9. I am confident I would recognize a friend on the phone, even if they were attempting to disguise their voice
10. I find it hard to identify which celebrities are voicing characters in animated films
11. When I overhear familiar people speaking, I know who they are before I see them
12. It is difficult to follow radio shows and podcasts with multiple speakers because their voices sound similar
13. I would notice immediately if a voice actor changed midway through an animated series
14. I find it hard to imagine familiar voices (e.g. the voice of celebrities, friends, family)
15. I can recognize which musician is performing a new song just from their voice
16. My ability to recognize people from their voice is worse than that of most other people

Nb. Items with odd numbers were reverse scored.

### Statistical procedures

Simple within-subjects contrasts were conducted using Student’s paired-samples *t*-tests. Where we could assume equal sample variance, simple between-subjects contrasts were conducted using Student’s between-samples *t*-tests. Where we could not assume equal sample variance, we employed Welch’s *t*-test. Comparisons of data with non-normal distributions were performed using Mann–Whitney tests. Correlations were evaluated by calculating Spearman Correlation coefficients. In all cases, the associated *p*-values described are two-tailed.

Where possible, we report Cohen’s *d* as a measure of effect size, calculated using ESCI^76^. However, where we could not assume equal variance between groups, we report a modified version of Cohen’s *d* whereby the difference in means is expressed relative to the square root of the average variance of the two groups^77^. Confidence intervals for both versions of *d* were calculated based on noncentral *t* distributions^76^.

## Results

### Identification accuracy

Participants’ performance on the famous voice and face recognition tasks was quantified as the proportion of voices or faces that were correctly identified, having discarded trials featuring people that were not known to the participant by name. Analyses including these trials produced very similar results, and are presented in the supplementary materials. One trial in the face task was discarded from one DP participant because they reported that the image failed to appear on the screen.

**Figure 1:**
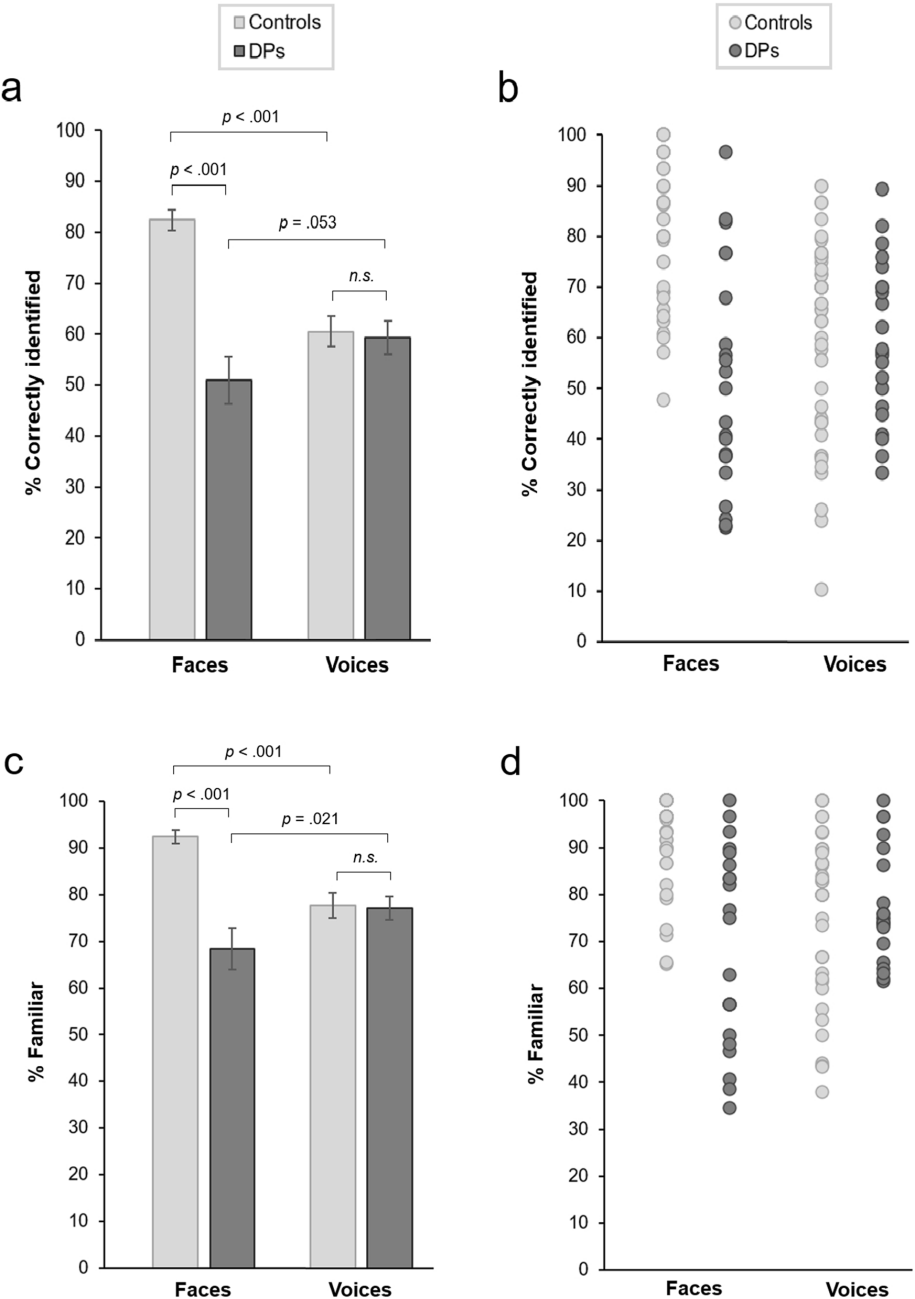
Performance in the famous face and voice recognition tasks. (**a**) Mean proportion of faces and voices that were correctly identified by the DP and control groups. (**b**) Identification accuracies for individual subjects. (**c**) Mean proportion of faces and voices that were classified as familiar by each group. (**d**) Familiarity scores for individual subjects. Error bars show standard errors.

Mean voice recognition performance was highly similar in DPs (*M* = 59.46%, *SD* = 15.61) and controls (*M* = 60.74%, *SD* = 19.52) [*t*(64) = 0.267, *p* = 0.791, *d* = 0.069, CI_95%_ = − 0.443, 0.581], suggesting that DPs show comparable famous voice recognition ability to typical controls (Fig. 1a,b). As expected, however, face recognition performance was significantly lower in DPs (*M* = 51.02%, *SD* = 21.77) compared with controls (*M* = 82.42%, *SD* = 13.55) [*t*(29.387) = 6.190, *p* < 0.001, *d* = 1.732, CI_95%_ = 0.951, 2.264].

ANOVA with Modality (faces, voices) as a within-subjects factor and Group (DPs, controls) as a between-subjects factor revealed a significant Modality × Group interaction [*F*(1,64) = 42.812, *p* < 0.001, η_p_^2^ = 0.401]. While controls recognised more faces than voices [*t*(43) = 8.584, *p* < 0.001, *d* = 1.267, CI_95%_ = 0.885, 1.686], DPs showed a non-significant trend to recognise more voices than faces [*t*(21) = 2.051, *p* = 0.053, *d* = 0.429, CI_95%_ = − 0.006, 0.887]. Sixteen of the 22 DPs (72.73%) recognised more voices than faces, compared with just 3 of 44 controls (6.82%). There were also significant main effects of Modality [*F*(1,64) = 8.272, *p* = 0.005, η_p_^2^ = 0.114] and Group [*F*(1,64) = 17.032, *p* < 0.001, η_p_^2^ = 0.210], reflecting better overall performance in the face task, and better overall performance of controls, respectively.

Analysis of the individual differences seen in the control sample revealed a significant correlation between participants’ face and voice recognition ability [*r*_*s*_ = 0.594, *p* < 0.001]. Despite the fact that their face recognition was worse overall, a similar association was seen in the DP sample [*r*_*s*_ = 0.537, *p* = 0.010]. However, it appears that this relationship reflects knowledge of popular culture (i.e., awareness of film, TV, sport, and current affairs). Typical participants who recognised more of the celebrities used in the voice task by name, tended to identify more of the famous faces [*r*_*s*_ = 0.455, *p* = 0.002]. This was also true of the DP sample [*r*_*s*_ = 0.559, *p* = 0.007]. Similarly, typical participants who recognised more of the celebrities used in the face task by name, tended to identify more of the famous voices [*r*_*s*_ = 0.570, *p* < 0.001], although this relationship was not significant for the DPs [*r*_*s*_ = 0.251, *p* = 0.259]. All correlations between identification performance, number of names reported as known, and perceived frequency of exposure for faces and voices, for the combined sample and for each group separately, are reported in the supplementary materials (Table S2).

### Perceived familiarity

Familiarity was expressed as the proportion of faces or voices that were classified as familiar (as opposed to unfamiliar), out of all trials featuring people that were subsequently recognised by name. Voice familiarity scores were highly similar for DPs (*M* = 77.14%, *SD* = 11.70) and controls (*M* = 77.70%, *SD* = 17.58) [*t*(58.668) = 0.153, *p* = 0.879, *d* = 0.037, CI_95%_ = − 0.472, 0.552] (Fig. 1c,d). In the famous face task, familiarity scores were significantly lower in DPs (*M* = 68.33%, *SD* = 20.66) compared with controls (*M* = 92.51%, *SD* = 9.49) [*t*(25.527) = 5.219, *p* < 0.001, *d* = 1.503, CI_95%_ = 0.721, 1.987]. ANOVA with Modality (faces, voices) as a within-subjects factor and Group (DPs, controls) as a between-subjects factor revealed a significant Modality × Group interaction [*F*(1,64) = 34.121, *p* < 0.001, η_p_^2^ = 0.348]. While controls found the faces more familiar than the voices [*t*(43) = 6.569, *p* < 0.001, *d* = 1.030, CI_95%_ = 0.661, 1.427], the DPs were more familiar with voices than faces [*t*(21) = 2.502, *p* = 0.021, *d* = 0.506, CI_95%_ = 0.097, 0.960]. There was no main effect of Modality [*F*(1,64) = 2.202, *p* = 0.143, η_p_^2^ = 0.033], and there was a significant main effect of Group [*F*(1,64) = 13.465, *p* < 0.001, η_p_^2^ = 0.174].

### Audio stimulus presentations

In the famous voice task, each clip could be played up to three times. To examine whether the results of the voice task were influenced by differential prioritisation of speed and accuracy, we examined how many times the two groups played the audio clips. Having averaged the number of presentations for each participant, we found that the median of the resulting distributions for the DPs (1.20) and controls (1.25) did not differ significantly [*U* = 438.5, *z* = − 0.62, *p* = 0.540]. The fact that the two groups played the audio clips a comparable number of times suggests a similar prioritisation of speed and accuracy by the DPs and controls.

### Name recognition and exposure frequency

When shown their names, both the DPs and the typical controls reported high levels of familiarity with the celebrities whose face or voice was used in the study. For the celebrities used in the face task, name recognition was similar for the DPs (*M* = 95.45%, *SD* = 6.71) and typical controls (*M* = 96.59%, *SD* = 6.41) [*t*(64) = 0.669, *p* = 0.506, *d* = 0.173, CI_95%_ = − 0.339, 0.687]. For the celebrities used in the voice task, name recognition was slightly lower for DPs (*M* = 92.43%, *SD* = 7.57) than for controls (*M* = 96.52%, *SD* = 5.75) [*t*(64) = 2.446, *p* = 0.017, *d* = 0.631, CI_95%_ = 0.113, 1.160]. This difference could be due to DPs and typical controls applying different criteria when asked whether they “know” a particular celebrity. For example, DPs may be less likely to say they “know” a celebrity if they have previously failed to recognise them, or are unsure of their ability to recognise them in the future.

**Figure 2:**
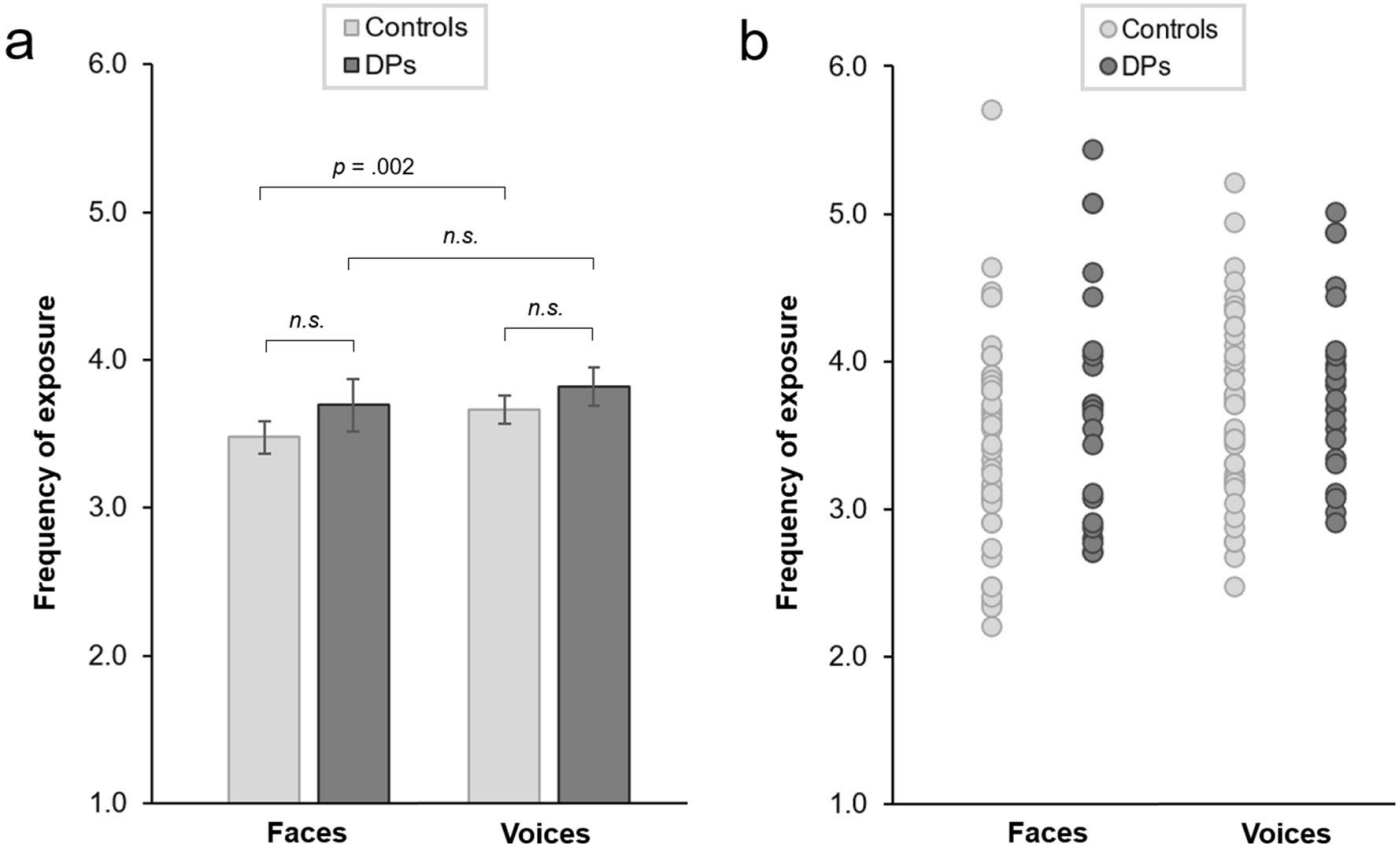
Ratings of perceived exposure frequency for the celebrities presented in the face and voice recognition tasks. (**a**) Mean ratings for faces and voices in the DP and control groups. (**b**) Individual subject ratings. Error bars show standard errors.

Ratings of exposure frequency were averaged across all voices used in the voice task, and all faces used in the face task, separately for each participant. Scores could range from 1 (‘never’) to 6 (‘very frequently’). Perceived frequency of exposure to faces was similar for DPs (*M* = 3.69, *SD* = 0.82) and controls (*M* = 3.48, *SD* = 0.71) [*t*(64) = 1.096, *p* = 0.277, *d* = 0.283, CI_95%_ = 0.229, 0.799] (Fig. 2). Despite DPs knowing fewer of the voice-task celebrities by name than controls, exposure to the voices was as frequent for DPs (*M* = 3.82, *SD* = 0.62) as it was for controls (*M* = 3.66, *SD* = 0.64) [*t*(64) = 0.980, *p* = 0.331, *d* = 0.253, CI_95%_ = − 0.259, 0.769]. For DPs, the frequency of exposure to the faces and to the voices did not differ significantly [*t*(21) = 1.345, *p* = 0.193, *d* = 0.168, CI_95%_ = − 0.087, 0.432]. Controls reported a slightly higher frequency of exposure to the voices than to the faces [*t*(43) = 3.376, *p* = 0.002, *d* = 0.263, CI_95%_ = 0.101, 0.432].

### Voice recognition questionnaire

**Figure 3:**
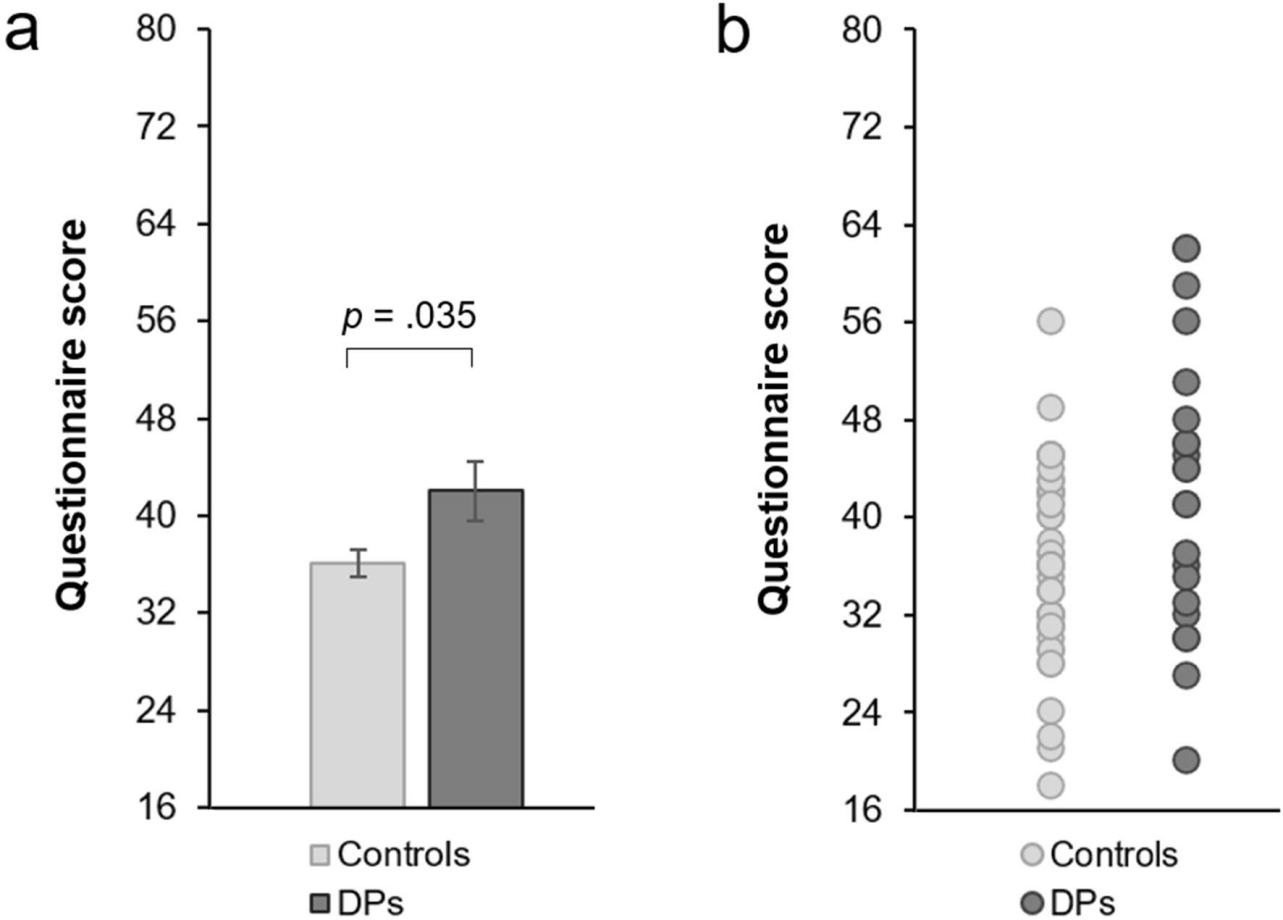
Scores on the voice recognition questionnaire. (**a**) Mean scores for the DP and control groups. (**b**) Individual subject scores. Higher scores indicate poorer perceived voice recognition ability.

Scores on the voice recognition questionnaire were higher for DPs (*M* = 42.05, *SD* = 11.42) than for controls (*M* = 36.09, *SD* = 7.76) [*t*(31.002) = 2.204, *p* = 0.035, *d* = 0.610, CI_95%_ = 0.040, 1.102] (Fig. 3). This contrasts with the finding of similar voice recognition performance in the task across the two groups, and suggests that DPs may have less confidence in their voice recognition ability. In the combined sample (*N* = 66) there was a small and non-significant correlation between questionnaire scores and voice identification performance [*r*_*s*_ = − 0.167, *p* = 0.180]. This was also the case in the DP [*r*_*s*_ = − 0.117, *p* = 0.604] and control [*r*_*s*_ = − 0.181, *p* = 0.240] groups separately.

## Discussion

In the present study we investigated the ability of individuals with DP to recognise famous faces and voices. As expected, DPs showed severely impaired recognition of famous faces relative to controls. In contrast, however, the performance of the DPs on the famous voice recognition task was very similar to that of typical controls. DPs not only identified a similar number of voices, they also judged a similar number of voices as familiar, when compared with controls. These findings cannot be explained by differences in familiarity and exposure to the celebrities’ faces and voices across groups.

Previous group studies of voice recognition in DP have used unfamiliar voices^57,58^. The results of these studies suggest that in most cases individuals with DP show typical discrimination and short-term memory for unfamiliar voices. Our results extend this literature by showing that DPs also perform typically when asked to identify well-known familiar voices. Importantly, our findings exclude the possibility of a selective vocal recognition deficit arising from the processing of person-related semantic information. Taken together, studies of familiar and unfamiliar voice identification suggest that DPs exhibit typical voice processing, and that their difficulties with person recognition are confined to the visual modality.

Evidence that face processing can be impaired independently from voice processing has implications for theoretical frameworks of person recognition, which propose that faces and voices are processed in hierarchical parallel pathways that interact with each other, and eventually converge for the post-perceptual processing of person identity^38,54–56,78–80^. The presence of a selective face deficit in DP suggests that despite evidence of interactions between face and voice identity processing^54–56^, there is some degree of dissociation between the two processing pathways, whereby one modality can be impaired while the other develops in a typical manner.

These findings also inform theoretical accounts of the origin and cause of DP. One possibility is that the condition arises from aberrant structure and function of multimodal regions such as the ATL. As a result, individuals with DP may struggle to retrieve person-related semantic information and benefit less from top-down contributions to face perception. However, a post-perceptual deficit affecting multi-modal regions would be expected to impede person recognition from both facial and vocal cues. The fact that DPs show typical voice recognition therefore argues against this account. Instead, these findings are more consistent with the view that DP is associated with an impairment early in the face processing stream that hinders the visual encoding of face structure^6,9,11,69^.

The absence of voice recognition deficits in DP suggests that previously observed abnormalities in the function and/or structure of multimodal brain regions in DP, in particular the ATL^50,51^ and the pSTS^52^, do not affect familiar voice processing. Although these regions are known to process identity from both faces and voices, it is likely that they are comprised of sub-regions that respond preferentially to faces, voices, or to both modalities^36,81^. Further neuroimaging work is needed to ascertain (i) whether DP selectively affects sub-regions dedicated to face processing, and (ii) whether aberrant structure and function of multimodal regions (pSTS and ATL) is a common feature of DP.

Our results support the claim that face and voice recognition ability are distinct from each other, rather than facets of a broader person recognition ability^82^. At first, this view seems hard to reconcile with the results of a recent study that found that individuals with exceptionally good face recognition ability—so called super-recognisers^83^—performed better than a group of typical controls on a famous voice identification task^84^. However, a close reading reveals that the super-recognisers in this study reported being more familiar with the celebrities whose voices were presented in the task than controls. The apparent association between face and voice recognition ability may also reflect the contribution of general factors such as motivation, attention, and familiarity with cognitive testing.

It has been demonstrated previously that people are much better at identifying celebrities based on their face than based on their voice^85–87^. This was evident in the better performance of our control sample on the famous face task, compared with the voice task. The DPs did not show this pattern; indeed, they showed signs of a voice recognition advantage. For example, they were more likely to find famous voices familiar, than famous faces. This is consistent with reports that DPs explicitly use the voice to identify familiar people when face identification fails^1^. However, while DPs may rely more on the voice for identification purposes, our results suggest that this doesn’t make them better at voice recognition compared to controls. In other words, the voice recognition pathway does not seem to compensate for a weak face recognition pathway in DP, potentially consistent with claims that the voice recognition pathway is inherently weaker^88,89^.

Despite performing as well as controls on the famous voice task, the DPs reported having worse voice recognition ability than controls on our self-report voice recognition questionnaire. Lifelong face recognition problems may cause individuals with DP to be circumspect about their relative ability in other domains. In some cases, confidence in non-face abilities may be further undermined by knowledge that DP can co-occur with non-face deficits including topographic agnosia^90^ and object agnosia^13,14,91^. In contrast, typical controls may have little or no reason to doubt their relative voice recognition ability. Where individuals take neurotypicality for granted—i.e., they underestimate neurodiversity in the population—they may over-estimate their relative ability in various domains.

Identification performance in the famous voice task was not correlated with performance on the voice recognition questionnaire. Similarly, a study employing a large sample of 730 participants, also found a very low correlation (*r* = 0.14) between performance on a famous voice recognition task and self-reported voice recognition ability^92^. It is possible that members of the general population have poor insight into their relative voice recognition ability. Indeed, the same study found that out of the 20 participants with the lowest scores on a famous voice test, only two reported below average voice recognition ability.

To summarise, the present study showed that individuals with DP exhibit intact familiar voice recognition ability, despite showing severely impaired recognition of famous faces. A possible explanation for this dissociation in DP could be that the deficit originates in the early perceptual encoding of face structure^6,9,11,69^, rather than at later, post-perceptual stages of face identity processing, which may be more likely to involve interactions with other modalities.

## Supplementary information

Supplementary Information.

## Data Availability

Data for the experimental tasks are available via the Open Science Framework (https://osf.io/da2xu/).
