# Supplementary material for: Normal recognition of famous voices in developmental prosopagnosia
Source: Sci Rep. 2020 Nov 12;10:19757. doi: 10.1038/s41598-020-76819-3 (PMC7661722; doi:10.1038/s41598-020-76819-3)
Supplement: Supplementary file 1 — Supplementary Information. [file 41598_2020_76819_MOESM1_ESM.pdf]

Normal recognition of famous voices in developmental prosopagnosia

Maria Tsantani and Richard Cook

Supplementary information

Identification accuracy unadjusted for name familiarity

In the main analysis, participants' performance in the famous voice and face recognition tasks was quantified as the proportion of voices or faces that were correctly identified, after discarding the trials featuring people that weren't known by name. Here, we report results including these trials.

Mean voice recognition performance was highly similar in DPs (M = 55.0%, SD = 15.46) and controls (M = 59.17%, SD = 20.33) [t(64) = .845, p = .401, d = .218, $CI_{95\%}$ = -.293, .733]. Face recognition performance was significantly lower in DPs (M = 49.52%, SD = 22.57) compared with controls (M = 80.45%, SD = 15.41) [t(31.103) = 5.788, p < .001, d = 1.601, $CI_{95\%}$ = .870, 2.137].

To investigate how famous face recognition ability compares to famous voice recognition ability in DPs and controls, we analysed performance on the face and voice tests in an ANOVA with Modality (faces, voices) as a within-subjects factor and Group (DPs, controls) as a between-subjects factor. There was a significant Modality × Group interaction [F(1,64) = 39.056, p < .001, η_p^2 = .379], driven by controls recognising more faces than voices [t(43) = 8.863, p < .001, d = 1.159, $CI_{95\%}$ = .816, 1.537], and a non-significant trend for DPs recognising more voices than faces [t(21) = 1.483, p = .153, d = .273, $CI_{95\%}$ = -.104, .664]. There were also significant main effects of Modality [F(1,64) = 13.619, p < .001, η_p^2 = .175] and Group [F(1,64) = 16.475, p < .001, η_p^2 = .205], driven by better overall performance in the face task, and better overall performance of controls, respectively.

Perceived familiarity unadjusted for name familiarity

Here we report results for judgements of perceived familiarity, without removing trials featuring people that were not subsequently recognised by name. Voice familiarity scores were similar for DPs (M = 73.79%, SD = 13.38) and controls (M = 76.21%, SD = 17.98) [t(64) = .559, p = .578, d = .144, $CI_{95\%}$ = -.367, .658]. In the famous face task, familiarity scores were significantly lower in DPs (M = 67.40%, SD = 21.53) compared with controls (M = 91.36%, SD = 11.55) [t(27.206) = 4.880, p < .001, d = 1.387, $CI_{95\%}$ = .653, 1.879]. ANOVA with Modality (faces, voices) as a within-subjects factor and Group (DPs, controls) as a between-subjects factor revealed a significant Modality × Group interaction [F(1,64) =

30.776, $p < .001$, $\eta_p^2 = .325$], driven by controls being familiar with more faces than voices [$t(43) = 7.048$, $p < .001$, $d = .985$, $CI_{95\%} = .649, 1.349$], and DPs tending to be familiar with more voices than faces, although the difference was not significant in a pairwise test [$t(21) = 1.867$, $p = .076$, $d = .343$, $CI_{95\%} = -.037, .741$]. There were significant main effects of Modality [$F(1,64) = 55.100$, $p = .027$, $\eta_p^2 = .074$] and Group [$F(1,64) = 12.533$, $p = .001$, $\eta_p^2 = .164$].

Table S1. Names of the celebrities presented in the face and voice recognition tasks.

Face recognition task		Voice recognition task	
Adele	Andy Murray	Cheryl Cole	Alan Carr
Victoria Beckham	Benedict Cumberbatch	Theresa May	Alan Rickman
Kate Middleton	David Cameron	Sharon Osbourne	Billy Connolly
Audrey Hepburn	Hugh Grant	Queen Elizabeth	Boris Johnson
Helena Bonham Carter	Tony Blair	Cilla Black	Stephen Fry
Maggie Smith	Jamie Oliver	Emma Watson	Sean Connery
Naomi Campbell	Colin Firth	Holly Willoughby	Nigel Farage
Olivia Colman	Daniel Craig	Jo Brand	Michael Caine
Keira Knightley	Simon Cowell	Joanna Lumley	David Attenborough
Margaret Thatcher	Gordon Ramsey	Judi Dench	John Cleese
Kate Winslet	Rowan Atkinson	Julie Waters	Jonathan Ross
Angelina Jolie	Bill Clinton	Mila Kunis	Barack Obama
Sandra Bullock	George W Bush	Dolly Parton	Will Smith
Meryl Streep	Matt Damon	Ellen DeGeneres	Tom Hanks

Table S2. Correlations (r_s) between different measures for both groups combined, and for DPs and controls separately.

		**Voices**			**Faces**		
		Ident.	Name fam.	Exp.	Ident.	Name fam.	Exp.
**Combined sample (N = 66)**							
Voices	Ident.	1.000	.297*	.320**	.476***	.484***	.228
	Name fam.		1.000	.443***	.567***	.571***	.294*
	Exp.			1.000	.364**	.471***	.817***
Faces	Ident.				1.000	.485***	.367**
	Name fam.					1.000	.478***
	Exp.						1.000
**DPs (N = 22)**							
Voices	Ident.	1.000	.041	.156	.537*	.251	.359
	Name fam.		1.000	.668**	.559**	.507*	.344
	Exp.			1.000	.493*	.609**	.747***
Faces	Ident.				1.000	.474*	.519*
	Name fam.					1.000	.666**
	Exp.						1.000
**Typical controls (N = 44)**							
Voices	Ident.	1.000	.395**	.386**	.594***	.570***	.183
	Name fam.		1.000	.436**	.455**	.574***	.343*
	Exp.			1.000	.544***	.435**	.857***
Faces	Ident.				1.000	.576***	.526***
	Name fam.					1.000	.405**
	Exp.						1.000

Nb. 'Ident.' refers to the proportion of faces/voice identified (including only celebrities who were known by name); 'Name fam.' refers to the number of names reported as known; 'Exp.' refers to the perceived frequency of exposure to the celebrities' faces/voices. *$p < .05$; **$p < .01$; *** $p < .001$.
